# Prognostic value of galectin-3 and right ventricular function for long-term mortality in heart failure patients treated with cardiac resynchronization therapy

**DOI:** 10.1038/s41598-021-00984-2

**Published:** 2021-11-01

**Authors:** Beata Zaborska, Ewa Pilichowska-Paszkiet, Ewa Makowska, Grażyna Sygitowicz, Tomasz Słomski, Michał Zaborski, Andrzej Budaj

**Affiliations:** 1 Department of Cardiology, Centre of Postgraduate Medical Education, Grochowski Hospital, Grenadierów 51/59, 04-073 Warsaw, Poland; 2 Department of Clinical Chemistry and Laboratory Diagnostics, Medical University of Warsaw, Warsaw, Poland; 3 SGH Warsaw School of Economics, Warsaw, Poland

**Keywords:** Biomarkers, Prognostic markers, Cardiology, Cardiac device therapy

## Abstract

Recently, associations between the biomarker galectin-3 and numerous pathological processes involved in heart failure (HF) and right ventricular (RV) function have been observed. We aimed to assess the long-term prognostic ability of galectin-3 and RV function parameters for all-cause mortality in HF patients treated with cardiac resynchronization therapy (CRT). We prospectively studied 63 symptomatic HF patients with a left ventricular (LV) ejection fraction (EF) ≤ 35%. The median serum galectin-3 concentration was 13.4 ng/mL (IQR 11.05, 17.15). A detailed assessment of LV and RV geometry and function was performed with echocardiography. CRT defibrillator implantation was achieved in all patients without major complications. The follow-up lasted 5 years. In the multivariable Cox regression model, independent predictors for all-cause mortality were log baseline galectin-3 and baseline RV function expressed as tricuspid annular plane systolic excursion with HR 2.96 (*p* = 0.037) and HR 0.88 (*p* = 0.023), respectively. Analysis of subgroups defined by galectin-3 concentration and CRT response showed that patients with high baseline galectin-3 concentrations and a lack of response to CRT had a significantly lower probability of survival. In our patient cohort, the baseline galectin-3 concentration and RV function were independent predictors of long-term all-cause mortality in HFrEF patients following CRT implantation.

## Introduction

Heart failure (HF) is a clinical syndrome with a high prevalence and remains one of the most challenging medical conditions. Moreover, HF is associated with high morbidity and mortality, reaching 50% at the 4-year follow-up, despite diagnostic and therapeutic efforts. Risk stratification remains difficult, even though numerous prognostic markers of death have been identified^1^. Therefore, there is a growing interest in biomarkers, both biochemical and imaging.

The biomarker galectin-3 (Gal-3), a lectin family member, is associated with numerous physiological and pathological processes in HF, including fibrogenesis, inflammation, and ventricular remodeling^2–4^. Galectin-3 is considered a “culprit” biomarker in HF, in contrast to “bystander” biomarkers such as N-terminal pro B-type natriuretic peptide (NT-pro BNP) or C reactive protein, as it stimulates pathological remodeling and the development of fibrosis, mainly by inducing fibroblast proliferation and collagen deposition^5^. Higher concentrations of Gal-3 have been found to be related to higher mortality in the general population and HF patients^2,6,7^.

Cardiac resynchronization therapy (CRT) has become a valuable, interventional therapeutic option for patients who, despite optimal medical therapy, have symptomatic HF with reduced ejection fraction (HFrEF). A few studies have shown data on the association between Gal-3 and adverse outcomes in CRT patients^8–10^. Excessive cardiac fibrosis and the presence of a myocardial scar were the likely causes of worse prognosis in patients with elevated Gal-3 concentrations. Right ventricular (RV) dysfunction, another potentially relevant mechanism, has been proven to be a prognostic factor among HF patients, including those treated with CRT^11,12^. Recently, some links between Gal-3 and RV function and pressure have been postulated. An association between Gal-3 and pulmonary artery hypertension was observed in experimental settings^13,14^. Furthermore, a few small studies have revealed the role of Gal-3 in RV remodeling and dysfunction induced by pulmonary arterial hypertension^14–16^. In our previous study in HFrEF patients, we found that Gal-3 was significantly negatively correlated with the echocardiographic parameter of long-axis RV function—tricuspid annular plane systolic excursion (TAPSE). Therefore, elevated Gal-3 concentrations in this population might indicate concomitant RV dysfunction^17^.

In the present study, we aimed to assess baseline Gal-3 and RV function parameters for the long-term prognosis of all-cause mortality in patients with HFrEF treated with a CRT defibrillator (CRT-D).

## Results

### Patient characteristics

Sixty-three symptomatic heart failure patients (81% males; mean age, 66.7 ± 8.9 years) in New York Heart Association (NYHA) class II or III, with a mean left ventricular (LV) EF of 25.6% (± 7.1%) and mean QRS duration of 156.8 ms (± 29.0 ms), were prospectively included. Seventy-three percent of them had ischemic cardiomyopathy. Five patients were upgraded from conventional RV apical pacing to biventricular pacing. The median serum Gal-3 concentration was 13.4 ng/mL (IQR 11.05–17.15 ng/mL).

### All-cause mortality

During the 5-year follow-up, 26 patients died. The median time from CRT-D implantation to death was 1.98 years (min:0.14, max:4.81 years). Four patients died within 6 months after CRT-D implantation. Table 1 shows a comparison of the study group's baseline demographic and clinical characteristics in relation to total mortality at 5 years. Significant differences in diastolic blood pressure, BMI and the history of dyslipidemia were found between the groups. In the survivor group, the concentrations of Gal-3, NT-pro BNP, and total bilirubin were lower than those in the nonsurvivor group. No significant differences were found in other parameters of clinical status, comorbidities, risk factors, ECG, hemoglobin level, platelet count, estimated glomerular filtration rate (eGFR), creatinine concentration or indexes of liver cirrhosis and fibrosis expressed as the Model for End-Stage Liver Disease eXcluding INR (MELD-XI) and Fibrosis-4 (FIB-4) scores, respectively.

**Table 1:** Baseline demographic and clinical characteristics of the patients in the whole group, nonsurvivor group and survivor group.

	All patients n = 63	Nonsurvivors n = 26 at 5-year FU	Survivors n = 37 at 5-year FU	*p*-value
Age (year)	66.7 ± 8.9	68.0 ± 8.8	65.7 ± 9.1	0.31
Sex (male/female), n (%)	51/12 (81/19)	24/2 (92/8)	27/10 (73/27)	0.06
Systolic blood pressure (mmHg)	120.2 ± 16.8	118.3 ± 17.8	121.6 ± 16.5	0.44
Diastolic blood pressure (mmHg)	73.9 ± 8.1	71.5 ± 5.3	75.5 ± 9.5	0.017
Body mass index (kg/m^2^)	28.0 ± 4.2	26.7 ± 3.6	28.9 ± 4.5	0.047
**NYHA class, n (%)**				0.18
II	26 (41)	9 (35)	17 (46)	
III	37 (59)	17 (65)	20 (54)	
**Etiology of heart failure, n (%)**				0.56
Ischemic	46 (73)	20 (77)	26 (70)	
Nonischemic	17 (27)	6 (23)	11 (30)	
Active smokers, n (%)	12 (19)	6 (23)	6 (16)	0.50
Former smokers, n (%)	19 (30)	8 (31)	11 (30)	0.93
**Past medical history, n (%)**				
Myocardial infarction	40 (63)	16 (62)	24 (65)	0.79
Hypertension	48 (76)	20 (77)	28 (76)	0.91
Diabetes mellitus	24 (38)	12 (46)	12 (32)	0.27
Dyslipidemia	27 (43)	4 (15)	23 (62)	< 0.001
**ECG rhythm**				0.34
Sinus rhythm	52 (83)	23 (88)	29 (78)	
Atrial fibrillation	6 (10)	1 (4)	5 (14)	
Paced (atrial pacing)	5 (8)	2 (8)	3 (8)	
ECG QRS duration (ms)	156.8 ± 29.0	154.6 ± 26.3	158.4 ± 31.4	0.48
**ECG QRS morphology**				0.40
LBBB	31 (49)	14 (54)	17 (46)	
RBBB	2 (3)	1 (4)	1 (3)	
IVCD	25 (40)	10 (38)	15 (41)	
Paced (RV pacing)	5 (8)	1 (4)	4 (11)	
Galectin-3 (ng/mL)*	13.4 (11.05, 17.15)	15.5 (12.8, 18.7)	12.1 (10.8, 14.8)	0.029
Hemoglobin (g/dl)	13.6 ± 1.6	13.3 ± 2.0	13.8 ± 1.2	0.16
Platelets (10^9^/L)*	209 (175, 257.5)	206 (164, 257)	226 (178, 256)	0.59
Creatinine (mg/dL)	1.28 ± 0.30	1.33 ± 0.32	1.25 ± 0.29	0.41
eGFR (ml/min/1.73 m^2^)	63.9 ± 24.4	61.1 ± 24.0	65.8 ± 25.1	0.54
Sodium (mmol/L)	139.7 ± 3.3	139.2 ± 4.2	140.0 ± 2.7	0.36
NT-pro BNP (pg/ml)* (p25–p75)	1549.0 (727.4, 2338.5)	1848.0 (1038.0, 3642.0)	1302.0 (496.0, 2216.0)	0.046
**Liver function**				
Total bilirubin (mg/dL)	1.13 ± 0.33	1.25 ± 0.38	1.05 ± 0.27	0.018
AST (U/L)	29.9 ± 17.7	29.7 ± 8.3	30.0 ± 22.4	0.10
ALT (U/L)	35.2 ± 22.8	35.5 ± 21.3	34.3 ± 24.2	0.35
INR	1.18 ± 0.29	1.20 ± 0.28	1.15 ± 0.30	0.07
**Scores**				
MELD-XI	12.4 ± 3.5	13.3 ± 3.3	11.7 ± 3.6	0.10
FIB-4, n (%)				0.41
< 1.45	28 (44)	10 (38)	18 (49)	
1.45–3.25	31 (49)	14 (54)	17 (46)	
> 3.25	4 (6)	2 (8)	2 (5)	
**Medications (%)**				
Diuretics	63 (100)	26 (100)	37 (100)	1.00
ACE inhibitors	52 (83)	21 (81)	31 (84)	0.76
ARBs	8 (13)	2 (8)	6 (16)	0.32
Beta-blockers	62 (98)	25 (96)	37 (100)	0.23
Antiarrhythmics	20 (32)	9 (35)	11 (30)	0.68
Aldosterone antagonists	44 (70)	19 (73)	25 (68)	0.64
Statins	51 (81)	19 (73)	32 (86)	0.19
Digoxin	7 (11)	2 (8)	5 (14)	0.47
Oral anticoagulants	18 (29)	9 (35)	9 (24)	0.38
**Response to CRT**				0.04
CRT responders	32 (51%)	10 (38)	22 (59)	
CRT nonresponders	27 (43%)	12 (46)	15 (41)	
Not assessed (death < 6 months)	4 (6%)	4 (15)	0 (0)	
Follow-up time (years)		1.98 (1.31–3.62)	5.0 (5.0,5.0)	NA

Values are expressed as the mean ± SD or counts and percent.

*Values are expressed as the median with interquartile range (p25–p75).

Gal-3, galectin-3; NYHA, New York Heart Association; LBBB, left bundle branch block; RBBB, right bundle branch block; eGFR, estimated glomerular filtration rate; NT-pro BNP, N-terminal pro B-type natriuretic peptide; AST, aspartate aminotransferase: ALT, alanine aminotransferase; INR, international normalized ratio; MELD-XI, Model for End-Stage Liver Disease eXcluding INR; FIB-4, Fibrosis-4; ACE inhibitors, Angiotensin-converting enzyme inhibitors; ARBs, angiotensin receptor blockers; CRT, cardiac resynchronization therapy.

No significant differences were found in medical therapy, although nonsurvivors tended to be treated with oral anticoagulants more frequently than survivors.

**Figure 1:**
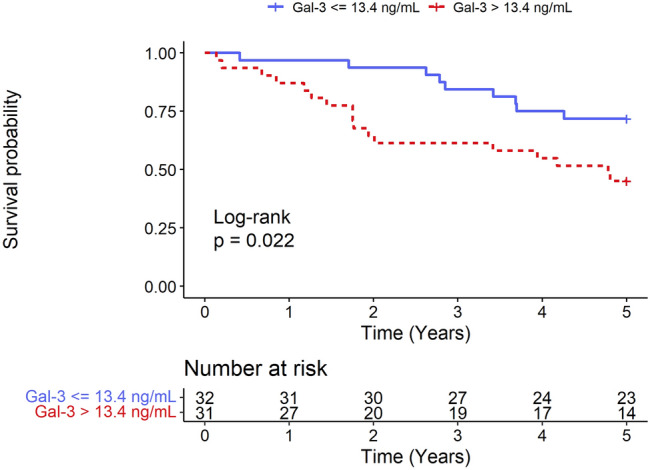
Title: Long-term survival following CRT-D according to baseline serum Gal-3 concentration. Kaplan–Meier survival estimates according to the baseline Gal-3 concentration dichotomized by a 13.4 ng/mL threshold. Patients with baseline Gal-3 concentrations above the median, 13.4 ng/mL, had a significantly lower probability of survival. Gal-3: galectin-3; CRT-D: cardiac resynchronization therapy-defibrillator.

Kaplan–Meier curves illustrate the cumulative survival of patients with low baseline concentrations of Gal-3 and those with high baseline concentrations of Gal-3 (Fig. 1). Patients with baseline Gal-3 concentrations above the median, 13.4 ng/mL, had a significantly lower survival probability. A log-rank test for equality of survivor function was performed with a *p*-value of 0.022.

In unadjusted Cox proportional hazards analysis, an increased hazard of death was found for the Gal-3 concentration (HR per log increase 4.44; 95% CI: 1.50–13.10, *p* = 0.007).

Table 2 shows a comparison of cardiac structure and function described by baseline echocardiographic measurements in relation to total mortality at five years. The association between mortality and baseline RV function was investigated. The group of nonsurvivors had significantly larger right ventricles and worse RV systolic and diastolic function parameters than the survivor group. Moreover, the RV-to-pulmonary circulation coupling-TAPSE/pulmonary artery systolic pressure (PASP) ratio, a noninvasive index, was significantly lower in nonsurvivors.

**Table 2:** Comparison of cardiac structure and function described by baseline echocardiographic measurements in the whole group, nonsurvivor group and survivor group.

Variables	All patients n = 63	Nonsurvivors n = 26 at 5-year FU	Survivors n = 37 at 5-year FU	*p*-value
RV inflow diameter (cm)	4.0 ± 0.7	4.1 ± 0.7	3.9 ± 0.7	0.15
RV outflow diameter (cm)	3.2 ± 0.5	3.3 ± 0.5	3.0 ± 0.5	0.025
RV sʹ (cm/s)	10.1 ± 2.7	9.1 ± 3.1	10.9 ± 2.9	0.034
RV eʹ (cm/s)	8.1 ± 3.3	7.0 ± 2.1	8.9 ± 3.3	0.027
TAPSE (mm)	19.2 ± 4.5	17.2 ± 4.4	20.6 ± 4.2	0.003
RV fractional area change (%)	38.7 ± 13.1	33.4 ± 12.8	42.5 ± 12.3	0.007
RV 2D strain (%)	− 13.2 ± 4.2	− 11.9 ± 3.6	− 14.0 ± 4.5	0.034
RV free wall 2D strain (%)	− 16.9 ± 5.8	− 15.1 ± 4.9	− 18.2 ± 6.2	0.07
TAPSE/PASP	0.71 ± 0.35	0.60 ± 0.36	0.80 ± 0.33	0.027
LV ejection fraction (%)	25.6 ± 7.1	24.7 ± 7.2	26.2 ± 7.1	0.42
LV end-diastolic volume (ml)	207.2 ± 59.1	217.7 ± 58.5	199.8 ± 60	0.24
LV end-systolic volume (ml)	157.0 ± 55.6	167.1 ± 56.3	149.9 ± 55.5	0.12
dP/dt (mmHg/s)	543.4 ± 141.0	514.3 ± 128.5	563.9 ± 149.2	0.22
LV sʹ (cm/s)	4.2 ± 1.2	4.1 ± 1.1	4.2 ± 1.4	0.92
LV GLS (%)	− 7.2 ± 2.6	− 6.9 ± 2.9	− 7.4 ± 2.5	0.15
**Mitral regurgitation, n (%)**				0.16
None/mild	35 (56)	12 (46)	23 (62)	
Moderate	21 (33)	10 (38)	11 (30)	
Severe	7 (11)	4 (15)	3 (8)	
**Tricuspid regurgitation, n (%)**				0.23
None/mild	47 (75)	18 (69)	29 (78)	
Moderate	15 (24)	8 (31)	7 (19)	
Severe	1 (2)	0 (0)	1 (3)	
LV E/eʹ	16.8 ± 7.2	18.9 ± 8.3	15.3 ± 6.1	0.11
PASP (mmHg)	33.7 ± 17.0	37.7 ± 19.3	30.8 ± 15.1	0.27
RAP (mmHg)	5.7 ± 4.3	6.5 ± 5.1	5.1 ± 3.6	0.31
LAVi (ml/m2)	44.8 ± 14.4	48.3 ± 17.3	42.4 ± 11.8	0.20
**LV diastolic dysfunction, n (%)**				0.30
Grade 1	27 (47)	10 (38)	17 (46)	
Grade 2	13 (23)	6 (23)	7 (19)	
Grade 3	17 (30)	9 (35)	8 (22)	

Values are expressed as the mean ± SD or counts and percent.

RV, right ventricular; sʹ, systolic myocardial velocity; eʹ, early diastolic myocardial velocity; TAPSE, tricuspid annular plane systolic excursion; PASP, systolic pulmonary artery pressure; LV, left ventricular; GLS, global longitudinal strain; E/eʹ, ratio of early diastolic transmitral velocity to peak early diastolic myocardial velocity; PASP, systolic pulmonary artery pressure; RAP, right atrial pressure; LAVi, left atrial volume index.

LV diastolic dysfunction assessment was performed for patients in sinus rhythm.

None of the baseline LV function or structure parameters differed significantly between survivors and nonsurvivors.

**Figure 2:**
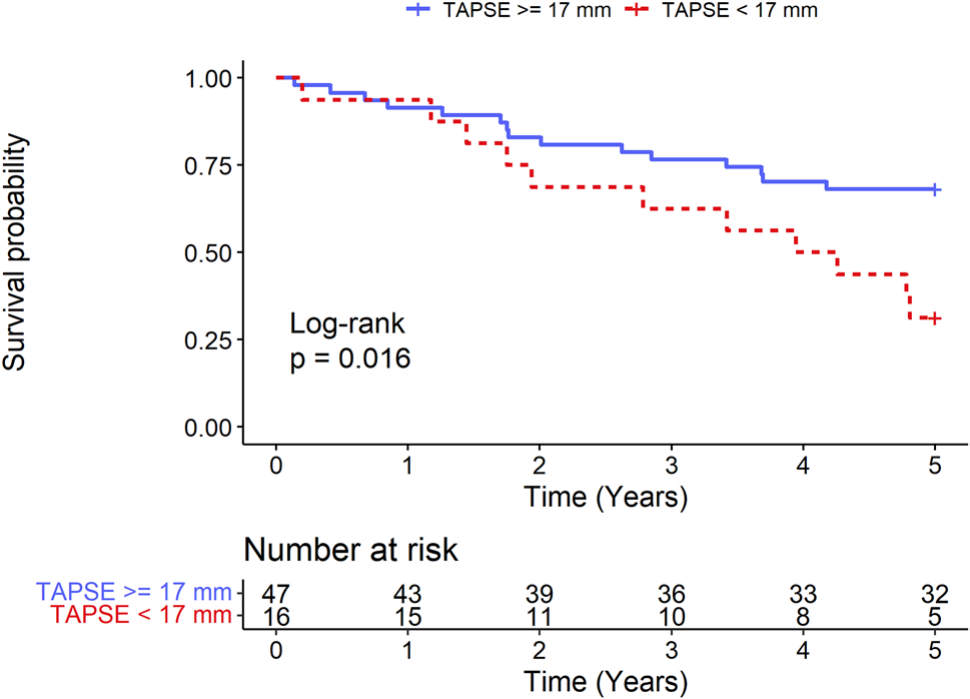
Title: Long-term survival following CRT-D according to RV function. Kaplan–Meier survival estimates according to baseline RV dysfunction defined as TAPSE under 17 mm. Patients with baseline TAPSE under 17 mm had a significantly lower probability of survival. CRT-D: cardiac resynchronization therapy-defibrillator; RV: right ventricular.

The association between baseline RV dysfunction, expressed by TAPSE under 17 mm, and long-term mortality was investigated. Kaplan–Meier curves illustrated the cumulative survival of patients with TAPSE under 17 mm and TAPSE equal to or over 17 mm (Fig. 2). Patients with a baseline TAPSE under 17 mm had a significantly lower survival probability. A log-rank test for equality of survivor function was performed with a p-value of 0.016. In unadjusted Cox proportional hazards analysis, a lower risk of death was found for TAPSE (HR per 1 mm increase 0.86; 95% CI: 0.78–0.95, *p* = 0.004).

### Response to CRT

Separate analysis was performed to examine the association of Gal-3 with long-term mortality taking into account the response to CRT-D implantation. CRT response, defined as a decrease of more than 15% in baseline LV end-systolic volume (LVESV), was assessed for 59 patients 6 months after CRT-D implantation. In four patients, cardiovascular death occurred before this time. Thirty-two (51%) patients were considered responders. The rate of CRT-D responders was higher in the survivor group; the data are shown in Table 1.

**Figure 3:**
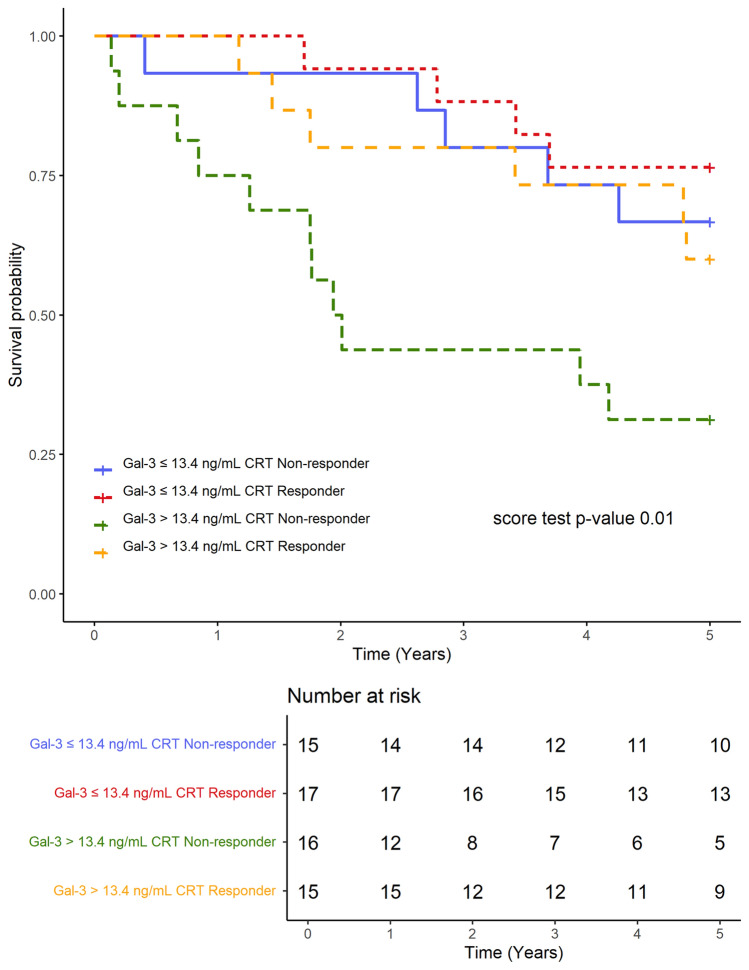
Title: Long-term survival following CRT-D according to the baseline Gal-3 concentration and CRT-D response. CRT-D: cardiac resynchronization therapy-defibrillator; Gal-3: galectin-3.

In the Cox regression score test, survival for four subgroups, defined by Gal-3 concentration and CRT response, differed significantly with a *p*-value of 0.01, which means that at least one of the four groups had a significantly different mortality rate than the others. The most unfavorable outcome was shown for patients with high Gal-3 concentrations and a lack of response to CRT (Fig. 3). Pairwise comparisons using the log-rank test with Bonferroni adjustment were performed between subgroups; the results are shown in Table 3.

**Table 3:** Pairwise comparisons using the log-rank test with Bonferroni adjustment performed between groups defined by baseline Gal-3 concentration and CRT response.

Log rank *p*-value	Gal-3 ≤ 13.4 ng/mL non-CRT responder	Gal-3 ≤ 13.4 ng/mL CRT responder	Gal-3 > 13.4 ng/mL non-CRT responder
Gal-3 ≤ 13.4 ng/mL CRT responder	0.55	–	–
Gal-3 > 13.4 ng/mL non-CRT responder	0.026	0.005	–
Gal-3 > 13.4 ng/mL CRT responder	0.74	0.32	0.065

The interaction between Gal-3 and the CRT response was tested in a Cox model with interaction. No significant interaction was found with an HR of 0.71 (95% CI 0.07–6.51, *p* = 0.76).

### Predictors of all-cause mortality; multivariable analysis

In Cox proportional hazards univariate analyses, the potential influence of covariates, namely, age, sex, HF etiology, LBBB, LVEF, log NT-pro BNP, eGFR, the MELD XI score, and CRT-D response, was assessed (Table 4). Variables with p-values below 0.1 were considered significant and were incorporated into multivariable Cox regression analyses using the backward elimination method. TAPSE and log Gal-3 remained significantly associated with all-cause mortality at the given time (Table 5). The HR for log Gal-3 for predicting death was 2.96 (95% CI 1.07–10.61, *p* = 0.037). RV function expressed as TAPSE was another independent predictor of long-term mortality, with an HR of 0.88 (95% CI 0.79–0.98, *p* = 0.023). In addition to log Gal-3 and TAPSE, the parameter of being a CRT responder was included in the model (model 1) of mortality prediction with an HR of 0.51 (95% CI 0.79–0.98, *p* = 0.1). After adjustment for age and sex (model 2), log Gal-3 and TAPSE remained significant independent predictors of all-cause mortality (Table 5). The Harrell’s C-Index reached a value of 0.732 for model 1 and 0.740 for model 2, which indicated that the risk score predictions were significantly better than the random classifier in determining which patients will live longer.

**Table 4:** Cox proportional hazards univariate analyses to identify predictors of death.

Predictors	Univariate	
	HR (95%CI)	*p*-value
Age	1.02 (0.98–1.07)	0.34
Male sex	3.16 (0.75–13.41)	0.12
Ischemic etiology	1.34 (0.54–3.35)	0.52
LBBB	1.31 (0.61–2.84)	0.49
LVEF	0.98 (0.93–1.03)	0.50
TAPSE	0.86 (0.78–0.95)	0.004
Log NT-pro BNP	1.67 (1.07–2.61)	0.03
Log Gal-3	4.44 (1.50–13.10)	0.007
eGFR	0.99 (0.98–1.01)	0.47
MELD XI	1.11 (0.99–1.25)	0.072
CRT responder	0.49 (0.22–1.08)	0.077

HR, hazard ratio; CI, confidence interval.

LBBB, left bundle branch block; Gal-3, galectin-3; TAPSE, tricuspid annular plane systolic excursion; NT-pro BNP, N-terminal pro B-type natriuretic peptide; eGFR, estimated glomerular filtration rate; LVEF, left ventricular ejection fraction; MELD-XI, Model for End-Stage Liver Disease eXcluding INR; CRT, cardiac resynchronization therapy.

**Table 5:** Multivariable Cox proportional hazards regression analyses to identify predictors of death.

Predictors	HR (95%CI)	*p*-value
**Model 1**		
Log Gal-3	2.96 (1.07–8.19)	0.037
TAPSE	0.88 (0.79–0.98)	0.023
CRT responder	0.51 (0.23–1.14)	0.10
**Model 2**		
Log Gal-3	3.68 (1.26–10.76)	0.017
TAPSE	0.89 (0.80–0.99)	0.035
CRT responder	0.50 (0.22–1.14)	0.10
Male sex	3.00 (0.33–12.98)	0.14
Age, y	1.04 (0.98–1.09)	0.19

HR, hazard ratio; CI, confidence interval.

Gal-3, galectin-3; TAPSE, tricuspid annular plane systolic excursion; CRT, cardiac resynchronization therapy.

## Discussion

This prospective, observational study demonstrated the association between the Gal-3 concentration and all-cause mortality in HFrEF patients treated with CRT and followed for 5 years. Gal-3 > 13.4 ng/mL was a predictor of worse prognosis. Of note, the Gal-3 concentration retained its independent predictive value for unfavorable outcomes regardless of CRT response. Our study confirmed the value of RV dysfunction as an independent predictor of long-term all-cause mortality after CRT implantation.

Galectin-3 is the only chimera-type member of the galectin family involved in numerous physiological and pathological processes and is crucial to processes involved in the development and progression of HF, such as fibrosis, inflammation, and remodeling^2–4^. Gal-3 significantly increases macrophage migration and resting fibroblast activation and promotes fibroblast proliferation. Moreover, Gal-3 is upregulated in hypertrophic hearts. It leads to increased type I collagen production and, as a consequence, increases myocardial rigidity^4^. As shown in several studies in animal or experimental models, the concentration of Gal-3 appeared to be a potent mediator of fibrosis^4,18^. Toprak et al.^3^ reported a possible role of increased Gal-3 concentration in the dilatation of cardiomyocytes and therefore tissue remodeling.

Several studies have already indicated that Gal-3 in HF could be a prognostic marker for mortality and rehospitalization^2,6,19–21^. However, Srivatsan et al.^22^, in a systematic overview, concluded that the current weight of the evidence did not suggest that Gal-3 is a predictor of all-cause mortality when factors such as renal failure, NT-pro BNP and LVEF were taken into consideration. The same authors acknowledged the need to study the utility of Gal-3 as a cardiac biomarker and called for research to explore its role.

Fibrosis and scar formation, which are maladaptive responses to injury or inflammation, are linked to disease progression and poor prognosis; therefore, they are relevant determinants of clinical outcome in HF^10,23^. The role of Gal-3 and other biomarkers of myocardial fibrosis in HF patients with CRT has been investigated in a few studies. However, the heterogeneity of the studied populations and echocardiographic methods, as well as various and combined endpoints, suggest the need for further studies. To our knowledge, this is the first study with death as the only hard endpoint in long-term follow-up. In the CARE-HF trial, serial changes in collagen turnover biomarkers including Gal-3 and the effects of CRT on these markers over an 18-month period were examined^9^. The study showed that CRT did not lead to significant changes in Gal-3 concentrations during long-term follow-up, suggesting that extracellular cardiac remodeling may not be involved in the beneficial effect of CRT. Nevertheless, the baseline serum Gal-3 concentration was associated with death or hospitalization for worsening HF at 18 months. This association was independent of NT-proBNP concentration, although it became nonsignificant after adjusting for renal function expressed as the value of eGFR^9^. In the MADIT-CRT trial, in which 654 patients with mild HF symptoms were randomized to CRT-D or implantable cardioverter defibrillator (ICD), an elevated Gal-3 concentration was a significant and independent predictor of nonfatal HF events or death^8^. However, in the CRT-D subgroup, an association of the Gal-3 concentration with outcome was absent^8^. Andre et al*.*^10^ confirmed the role of Gal-3 in predicting the response to CRT and long-term outcomes defined as death and hospitalization for a major adverse cardiovascular event (MACE) composite of hospitalization for HF, cardiogenic shock and sustained ventricular tachycardia. They also showed that Gal-3 serum concentrations equal to or greater than 22 ng/mL predicted survival after CRT implantation at the 48-month follow-up. However, they studied a particular patient population since only patients with typical left bundle branch block (LBBB) were included in the study. The response to CRT, defined as reversed remodeling expressed as a reduction in LV end-systolic volume, was found to be a strong predictor of reduced all-cause long-term mortality^24^. In our study group, the most unfavorable outcome was observed for CRT nonresponders with high Gal-3 concentrations. Interestingly, there was no significant difference between responders with low Gal-3 concentrations and nonresponders with low Gal-3 concentrations.

RV function was recognized as a significant prognostic marker in HFrEF patients in pathophysiological models of ischemia and dilated cardiomyopathy^25,26^. This finding was also confirmed in some studies for HF patients treated with CRT^11,12^. Leong et al.^11^ found that baseline RV impairment, quantified by TAPSE as a highly feasible echocardiographic measure, was an independent predictor of all-cause mortality following CRT and conferred increased prognostic value over a broad range of clinical and echocardiographic parameters. In the cited study, RV dysfunction was observed in 34% of individuals. This is in concordance with our finding that TAPSE was an independent prognostic marker of long-term all-cause mortality. Echocardiographic diagnosis of RV function is complex and challenging. Therefore, various parameters have been used and examined for prognosis. Sade et al.^12^ demonstrated that preserved RV function—assessed by a novel echocardiographic application, speckle tracking strain—was an independent predictor of long-term event-free survival after CRT. In our study, RV dilatation, a decline in RV long-axis systolic function and RV strain impairment were observed in nonsurvivors at baseline echocardiographic examination. All the findings mentioned above are elements of a vicious circle in HFrEF when volume and pulmonary pressure overload lead to RV dysfunction development. Uncoupling of the right ventricle-pulmonary artery, expressed as significantly lower TAPSE/PASP ratios, was also found. Recently, the association between TAPSE/PASP and the established markers of prognosis NT-pro BNP and E/e´ ratio were found in patients undergoing CRT^27^.

Thus, existing research has demonstrated the prognostic value of RV dysfunction for all-cause mortality. Importantly, it has yet to be demonstrated whether the prognostic ability of RV function assessment is confounded by other important determinants of outcome, such as a markers of fibrosis. In the present study, multivariate analysis revealed that the concentration of Gal-3 was an independent prognostic marker along with RV dysfunction expressed as TAPSE. In our previous study, we found that in symptomatic patients with chronic HFrEF, an elevated Gal-3 concentration was related to worsened RV function but not LV function. Significant, negative, moderate correlations between the Gal-3 concentration and RV long-axis function parameters (TAPSE and RVs’) as well as TAPSE/PASP were observed, and a moderate, significant, positive correlation between Gal-3 and PASP was found^17^^.^

The development of RV dysfunction in HF patients may result from various pathological mechanisms, including RV injury, remodeling, fibrosis, and pulmonary hypertension. Recently, potential mechanisms underlying the relationship between Gal-3 and RV function have been investigated and reported. Established right ventricular fibrosis in patients with pulmonary hypertension and in experimental animal models was found to be characterized by marked expression of Gal-3 and enhanced numbers of proliferating RV fibroblasts^28^. Furthermore, He et al.^15^, in a study on patients with pulmonary artery hypertension and in an animal model, found that Gal-3-mediated pulmonary artery hypertension through NADPH oxidase 4 and NADPH oxidase 4-derived oxidative stress led to RV remodeling. A facilitating role of Gal-3 in pulmonary artery remodeling and the progression of pulmonary artery hypertension were reported by Shen et al.^14^ in patients with congenital heart disease. In an animal model, Hao et al.^13^ showed that Gal-3 inhibition ameliorated hypoxia-induced pulmonary artery hypertension and reduced the inflammatory response. Although patients with primary pulmonary hypertension were not represented in our study group, we observed nonsignificantly higher PASP and right atrial pressure (RAP) and a significantly lower TAPSE/PASP ratio in the nonsurvivor group. Recently, the TAPSE/PASP ratio was found to be an important prognostic marker of mortality in HFrEF patients^29^.

### Limitations

Certain limitations of this study should be considered. First, this is a single-center study with a relatively small number of patients. Second, advanced HFrEF is an extremely complex syndrome with multiorgan involvement and requires multiparameter assessment. We were able to analyze a limited number of parameters, but not all potentially relevant parameters, in multivariate analyses. Third, hospitalization for HF was not defined as an outcome in our study. We chose all-cause mortality as the only hard endpoint, and therefore, we avoided the risk of error in the adjudication of events. We are aware that Gal-3 is a potent marker of fibrosis found in a wide range of species and tissues. In addition, it plays a regulatory role in inflammation and cancer. Gal-3 might have an impact on all-cause mortality regardless of advanced heart failure. To our knowledge, three patients died due to lung cancer. However, we did not include patients with chronic inflammatory diseases. Although we used multiple echocardiographic techniques to asses RV parameters, including advanced speckle tracking analyzes, it may be not as accurate as 3D echocardiography, which allows better RV volumes and function characterization^30^. However, we did not have access to 3D echocardiography at the time of the study.

## Conclusion

This study indicates that elevated Gal-3 concentrations and RV dysfunction quantified by TAPSE are independent predictors of long-term all-cause mortality in HFrEF patients after CRT implantation. An association between the baseline Gal-3 concentration and outcome was found regardless of CRT response, which requires further research.

## Methods

### Study population and design

The design of this study and the initial results were previously described and published^17,31^. A total of 67 consecutive patients referred to our Cardiology Department for implantation of a CRT defibrillator (CRT-D) were included between January, 7 2013 and November, 28 2015 and prospectively assessed. The inclusion criteria were as follows: symptomatic heart failure in NYHA classes II–III despite optimal medical therapy, severe LV systolic dysfunction—EF ≤ 35%, and QRS duration ≥ 120 ms. Patients were qualified for CRT-D implantation by the attending cardiologist according to current ESC Guidelines for the diagnosis and treatment of acute and chronic heart failure^32^. Patients with hemodynamic instability, severe organic valvular disease, chronic lung disease, end-stage liver or kidney disease, or chronic inflammatory disease were excluded. The ischemic etiology of heart failure was proven by coronary angiography or by a documented history of myocardial infarction. At the time of qualification for CRT-D, none of the patients required surgical or interventional revascularization for coronary symptoms. Of these patients, 63 underwent CRT-D implantation and were able to participate in the follow-up.

The study was approved by the Bioethical Committee of the Centre of Postgraduate Medical Education and was performed following the requirements of the Declaration of Helsinki. All the participants signed informed consent before inclusion in the study.

Clinical evaluation with NYHA functional status assessment, ECG and transthoracic echocardiography (TTE) was performed within 48 h before CRT implantation. Blood samples were obtained from each patient, and routine laboratory tests, including measurements of serum NT-pro BNP concentrations, were performed immediately. The remaining serum was frozen for further Gal-3 concentration measurements. Even though patients with end-stage liver disease were not enrolled, to have a broader description of patient characteristics, we calculated indexes of cirrhosis and liver fibrosis using well-defined formulas of the following scores:

Model for End-Stage Liver Disease eXcluding INR (MELD-XI) = 5.11 × (ln of total bilirubin in mg/mL) + 11.76 × ln of creatinine in mg/mL) + 9.44;

Fibrosis-4 (FIB-4) = (age [years] x aspartate aminotransferase [U/L])/(platelets [10^9^/L] × alanine aminotransferase^1/2^ [U/L]^33,34^.

Transthoracic echocardiography was repeated 6 months after device implantation to assess the response to CRT.

### Echocardiographic assessment

Echocardiographic examinations were performed with a Vivid E9 ultrasound system (GE Healthcare, Horten, Norway) by experienced cardiologists, according to the recommendations of esteemed echocardiographic societies (American Society of Echocardiography, European Association of Echocardiography and Canadian Society of Echocardiography)^35,36^. All measurements were taken with regard to the current standards using M-mode echocardiography, 2D echocardiography, Doppler ultrasound, pulsed tissue Doppler and 2D speckle tracking, as previously described in detail and published elsewhere^17,31^.

The evaluation of the LV comprised assessment of geometry, systolic function expressed as EF (calculated according to modified Simpson's rule), dP/dt, LV peak systolic myocardial velocity (sʹ) and global longitudinal strain (GLS), diastolic function expressed as LV diastolic dysfunction grade, E/eʹ ratio, left atrial maximum volume index (LAVi), and mitral regurgitation grade.

Right ventricular evaluation comprised detailed geometric calculations and functional assessment: TAPSE, fractional area change (FAC), RV peak systolic myocardial velocity (sʹ), peak early (eʹ) diastolic velocity, RV 2D systolic speckle tracking-derived longitudinal strain, and free wall 2D strain. Based on tricuspid regurgitation, peak velocity and respiratory variation in inferior vena cava diameter, PASP and RAP were estimated. A TAPSE/PASP ratio was used to noninvasively assess RV-to-pulmonary circulation coupling.

### Galectin-3 concentration measurement

The concentration of serum Gal-3 was quantified using VIDAS (*bioMerieux SA*, France)^37,38^. Previously collected clinical samples were thawed at room temperature just before the measurement of the Gal-3 concentration. An enzyme-linked fluorescence assay (ELFA) was used to measure the serum Gal-3 concentration. This method is a combination of an immunoassay with a final fluorescence measurement. The functional measuring range of ELFA is between 3.3 and 100 ng/mL, and the assay has high repeatability (CV approximately 1%) and reproducibility (CV approximately 5%). The intra-assay variances for Gal-3 were 1.25%, and the interassay variances for Gal-3 were 5.5%.

### CRT device implantation

CRT-D device and lead placements were achieved in all patients without major complications. The LV pacing lead was implanted transvenously through the coronary sinus in lateral and posterolateral venous branches whenever possible. All the patients had biventricular pacing with atrioventricular (in patients with sinus rhythm) and interventricular delays optimized using Doppler echocardiography.

### Follow-up

All the patients were under the care of the outpatient CRT clinic during the follow-up period. Follow-up started on the date of CRT-D implantation and ended after 5 years or at the time of death. All-cause mortality was the endpoint of the study. Data on long-term all-cause mortality were collected, and complete data for each patient were obtained from the hospital database, outpatient clinic records and interviews with patients’ relatives.

The patients were classified as CRT-D responders if they showed a decrease equal to or greater than 15% in LV end-systolic volume at the 6-month follow-up TTE^39^.

### Statistical analyses

Continuous variables are presented as the mean ± standard deviation (SD) or as the median and interquartile range (IQR) when appropriate. Categorical variables are presented as numbers and percentages.

The normality of continuous parameters was tested using the Shapiro Wilk test. Then, two-sample t-tests and the Kruskal–Wallis test were used to test whether samples originated from the same distribution. A *p*-value < 0.05 was considered statistically significant.

Survival curves were calculated using the Kaplan–Meier method, and a log-rank test, or score test and log-rank test with Bonferroni adjustment respectively, were used for assessment overall differences between groups. The Cox proportional hazards model was used to identify independent predictors of long-term all-cause mortality and to assess the influence of potential confounding variables. After assessing the importance of the variables using a univariate Cox proportional hazards model, we selected further modeling variables with a *p*-value < 0.1. Then, using the backward elimination method, we obtained the final model (model 1), which was then adjusted for demographic variables (model 2). Model assumptions were tested on the basis of Schoenfeld residuals^40^. The natural logarithm of Gal-3 and the natural logarithm of NT-pro BNP were used in the Cox model to improve the linearity assumption. We used Harrell’s C-index (concordance index) to assess the models’ goodness of fit^41^. Statistical analysis was performed using R software, version 4.1.0, https://www.R-project.org/.

## Data Availability

The datasets generated and analyzed during the study are available from the corresponding author on reasonable request.
